# Single-stage laparoscopic transabdominal preperitoneal (TAPP) utilizing biologic mesh for De Garengeot hernia: a case report and literature review

**DOI:** 10.3389/fmed.2025.1643775

**Published:** 2025-08-21

**Authors:** Hong-Xia Song, Tian-Hao Xie, Yan Fu, Xiao-Shi Jin, Qiang Wang, Zheng Niu

**Affiliations:** ^1^Department of General Surgery, Affiliated Hospital of Hebei University, Baoding, Hebei, China; ^2^Basic Research Key Laboratory of General Surgery for Digital Medicine, Affiliated Hospital of Hebei University, Baoding, Hebei, China; ^3^Department of Ophthalmology, Baoding No.1 Central Hospital, Baoding, Hebei, China

**Keywords:** femoral hernia, TAPP, biologic mesh, case report, De Garengeot hernia

## Abstract

De Garengeot hernia, characterized by appendiceal incarceration within a femoral canal hernia sac, is a rare condition with high risks of strangulation. Traditional open repair remains standard, but laparoscopic approaches offer minimally invasive alternatives. However, limited evidence exists on combining laparoscopic transabdominal preperitoneal (TAPP) with biologic mesh for this condition. This study presents a case of laparoscopic TAPP with biologic mesh for De Garengeot hernia with concomitant laparoscopic appendectomy. A 69-year-old woman presented with a right inguinal mass and pain. Imaging confirmed a femoral hernia containing the inflamed appendix. Laparoscopic exploration revealed ischemic appendiceal changes, necessitating appendectomy. Laparoscopic TAPP with biologic mesh was performed, utilizing keyhole fixation and reinforced closure. Postoperative recovery was uneventful, as evidenced by no recurrence at 18-month follow-up. Laparoscopic TAPP with biologic mesh represents a feasible, minimally invasive strategy for De Garengeot hernia, enabling simultaneous appendectomy and hernia repair. This approach leverages the regenerative properties of biologic scaffolds and their superior anti-infective properties, while minimizing complications, offering a promising alternative to traditional methods. Further research is needed to establish standardized protocols and assess long-term outcomes.

## Introduction

De Garengeot hernia, first described in 1731, is a rare clinical entity characterized by the presence of the vermiform appendix within a femoral canal hernia sac, accounting for 0.15%–5% of femoral hernia cases (1). The narrow anatomical confines of this hernia predispose patients to incarceration and strangulation, thereby necessitating urgent surgical intervention. Historically, open repair with appendectomy and primary tissue closure has been the standard treatment (2). Although laparoscopic techniques have emerged as minimally invasive alternatives, their adoption as primary therapeutic strategies faces two critical barriers: (1) insufficient validation of procedural safety through robust clinical studies, and (2) unresolved uncertainties regarding preoperative diagnostic accuracy. Current evidence remains inadequate to establish laparoscopic methods as standardized protocols, as both technical specifications and clinical indications require further validation through large-scale studies (3). A systematic review by Gómez-Portilla et al. (3) identified 29 cases of De Garengeot hernia treated exclusively via laparoscopic approaches, predominantly through the transabdominal preperitoneal (TAPP) technique (*n* = 25) or totally extraperitoneal (TEP) repair (*n* = 4).

Biologic meshes confer potential advantages in contaminated surgical fields, including demonstrated anti-infective properties (4), the ability to promote tissue remodeling (5), and reduced recurrence rates relative to synthetic counterparts (6). Despite these attributes, their use in De Garengeot hernia repair remains exceedingly limited. To date, only two cases of hybrid procedures combining laparoscopic appendectomy with open biologic mesh reinforcement have been reported (7, 8). Notably, no published studies describe the application of biologic meshes in a fully laparoscopic TAPP approach. This case report describes the application of a biologic mesh in laparoscopic TAPP for a DeGarenge hernia combined with laparoscopic appendectomy. We analyze the technical feasibility of this approach, evaluate the rationale for biologic mesh selection, and discuss potential implications for clinical practice.

## Case presentation

**FIGURE 1:**
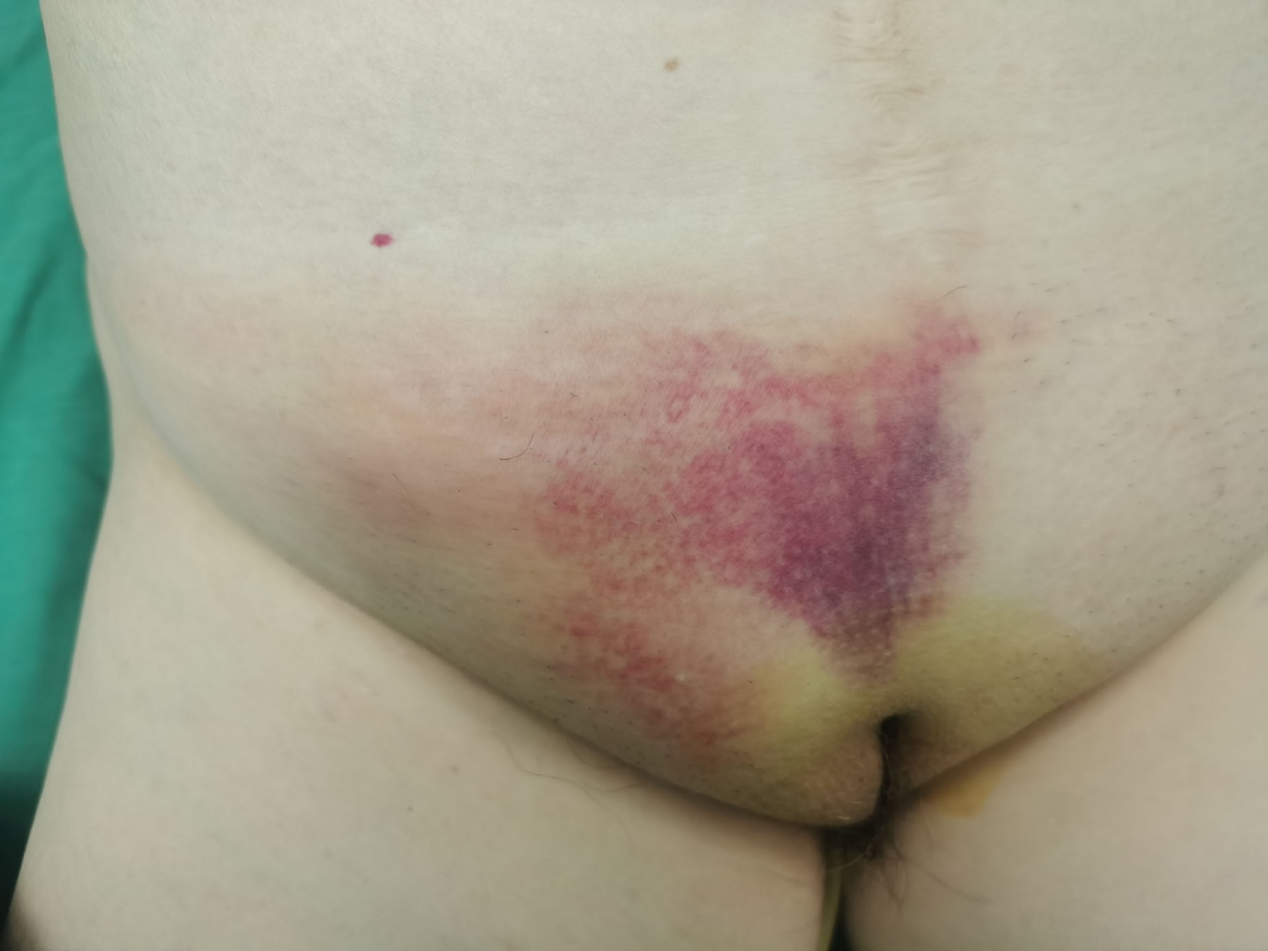
The right inguinal area demonstrated localized erythema, warmth, and a firm 3-cm irreducible tender bulge.

**FIGURE 2:**
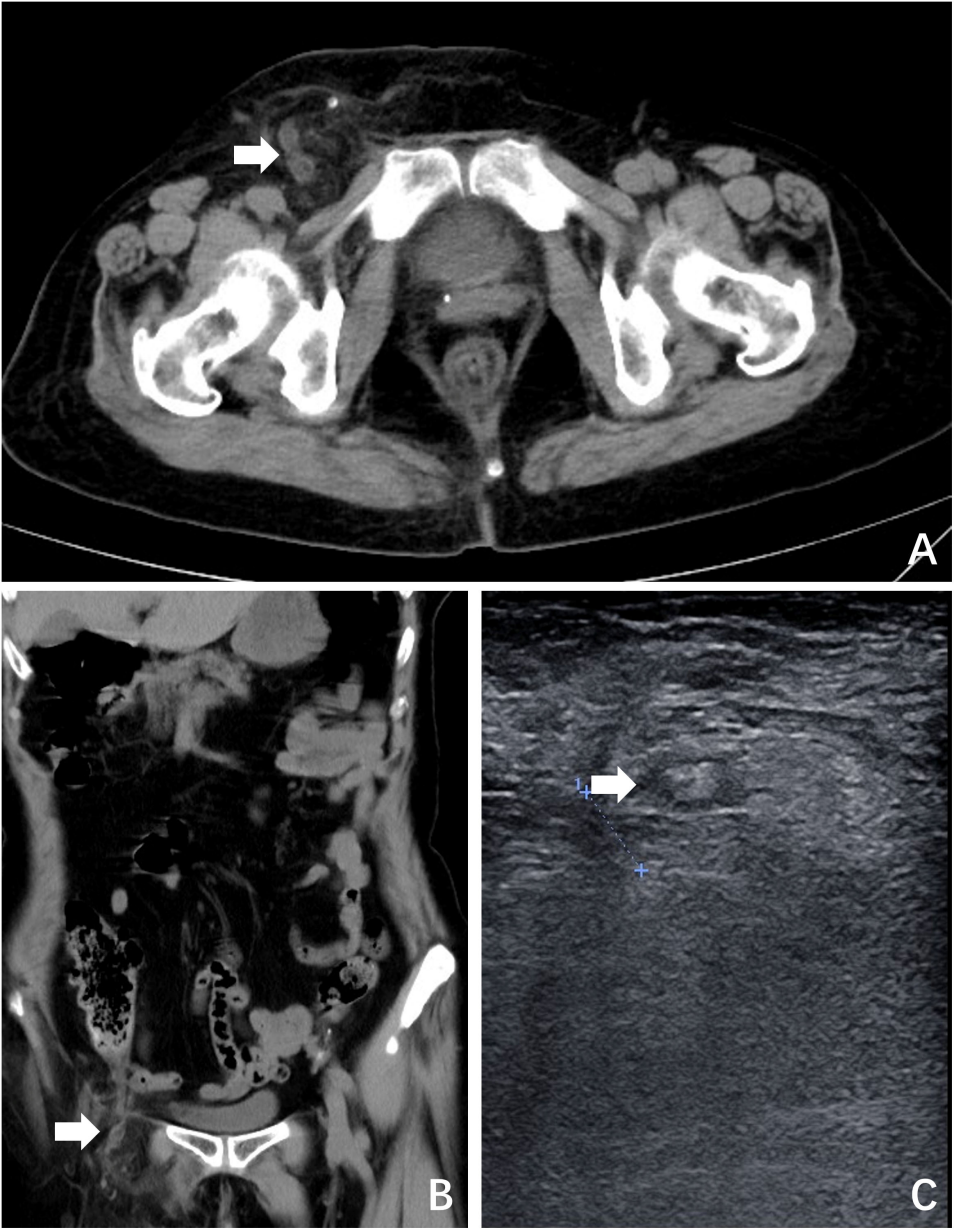
Preoperative imaging. **(A)** CT revealed a right femoral hernia defect inferior to the inguinal ligament and lateral to the pubic tubercle, containing the herniated appendix (white arrow); **(B)** coronal plane view of CT; **(C)** ultrasonography identified an incarcerated right femoral hernia containing the appendix (white arrow).

A 69-year-old woman presented with a 24-h history of right inguinal distension and pain. The patient developed a tender, irreducible bulge in the right inguinal region after heavy lifting in a forward-flexed position. She reported maintained flatus passage but denied vomiting, diarrhea, or febrile symptoms. Medical history included hysterectomy for uterine fibroids three decades prior, with no personal/family history of hernias or malignancies. Abdominal examination was unremarkable. The right inguinal area demonstrated localized erythema, warmth, and a firm 3-cm irreducible tender bulge (Figure 1). Computed tomography (CT) revealed a right femoral hernia defect inferior to the inguinal ligament and lateral to the pubic tubercle, containing the herniated appendix (Figures 2A, B). Concurrent ultrasonography identified an incarcerated right femoral hernia with a tubular structure in the sac consistent with appendiceal involvement, showing wall thickening, periappendiceal fat stranding, and increased attenuation (Figure 2C). Laboratory parameters (complete blood count, hepatic/renal function) remained within normal limits. The patient was diagnosed with De Garengeot hernia complicated by acute appendicitis.

**FIGURE 3:**
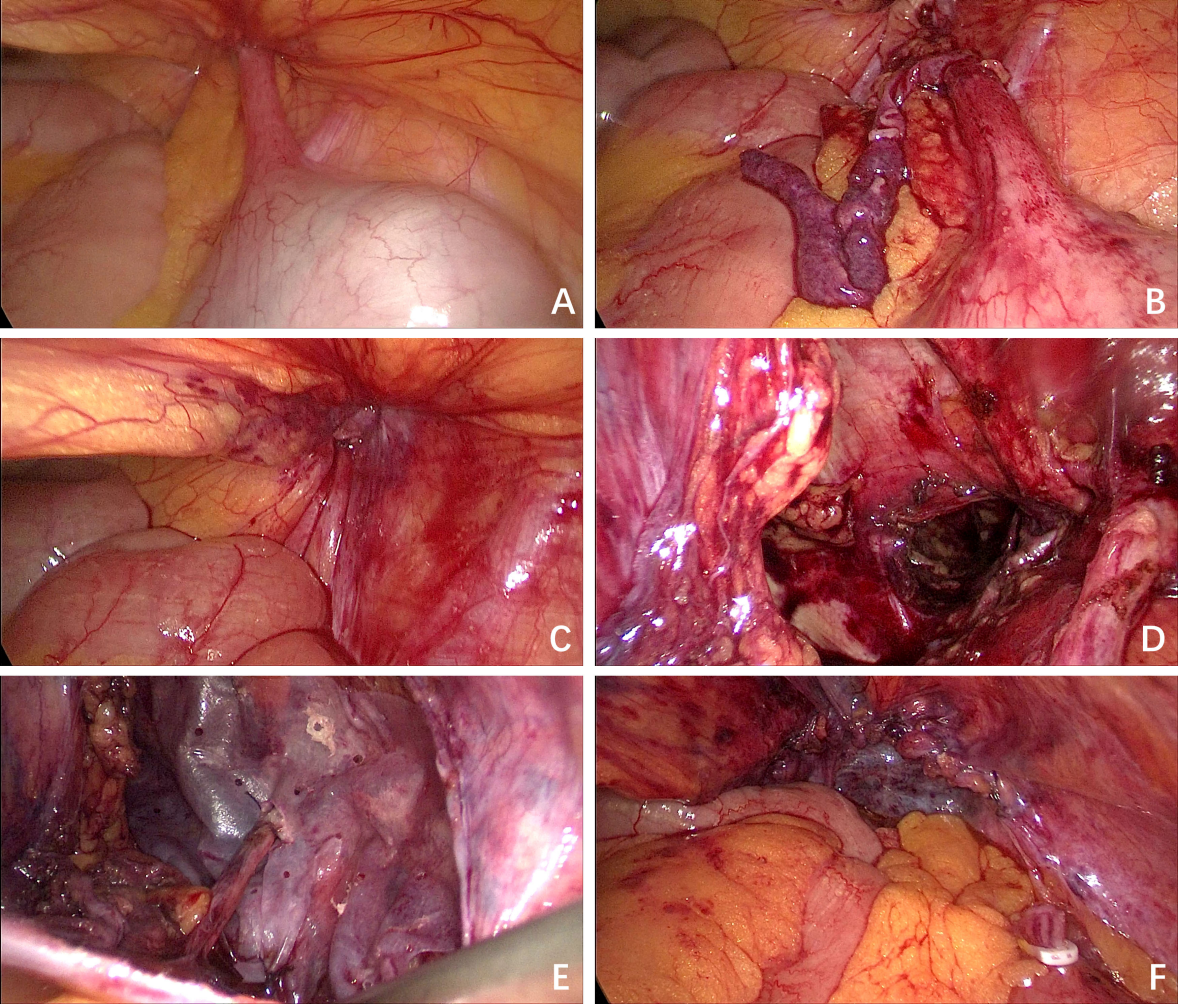
Intraoperative situation. **(A)** The appendix was found incarcerated within the femoral ring; **(B)** the appendix exhibited ischemic changes after reduction; **(C)** the defect of the femoral ring was observed; **(D)** the defect of the femoral ring after the hernia sac was completely reduced; **(E)** the biologic mesh was placed over the myopectineal orifice, and a keyhole technique was employed to encircle the round ligament; **(F)** the peritoneum was closed continuously with absorbable sutures.

Following informed consent, laparoscopic exploration was performed. The patient was placed in a 15° Trendelenburg supine position. A 10 mm incision was made at the superior aspect of the umbilicus to establish pneumoperitoneum and introduce a trocar, serving as the observation port. Intraoperatively, the distal appendix was found incarcerated within the femoral ring (Figure 3A). Subsequently, 10 mm and 5 mm incisions were made at the lateral borders of the right and left recti abdominis, respectively, for trocar placement as working ports. The 10 mm port was primarily used for the placement of Hem-o-lok clips. After reduction, the appendix exhibited ischemic changes (Figure 3B), necessitating appendectomy. A residual bulge in the right inguinal region, attributed to a thickened hernial sac, was palpated (Figure 3C). Given the absence of appendiceal perforation or peritonitis, a TAPP was conducted. The hernial sac was completely reduced (Figure 3D), and femoral hernia repair with biologic mesh reinforcement was performed.

The preperitoneal space dissection was extended 3 cm superior to the conjoined tendon, laterally to the psoas major muscle and the anterior superior iliac spine, medially to the pubic symphysis, inferomedially to 3 cm inferior to the pectineal (Cooper) ligament, and inferolaterally to include 8 cm of the round ligament of the uterus after its mobilization and fixation to the abdominal wall. This procedure ensured adequate exposure for mesh placement. The femoral ring was closed with absorbable sutures, followed by placement of a porcine small intestinal submucosa acellular matrix mesh (Surgisis^®^, Cook Medical, USA), specifically designed for TAPP, measuring 10 × 15 cm and positioned over the myopectineal orifice. A standardized keyhole technique was employed, involving the creation of a 5-mm diameter circular opening in the mesh to accommodate the round ligament. The mesh was secured to the transversalis fascia using intermittent 3-0 absorbable sutures, with tension-free fixation confirmed (Figure 3E). Sutures were loosely tied to prevent mesh displacement while avoiding entrapment of nerves in the pain triangle. A 5-mm trocar was inserted at a point 1 cm lateral to the mesh. Through this trocar, a 12-Fr drainage tube was positioned between the mesh and peritoneum, ensuring that the drainage tube and its lateral fenestrations traversed the preperitoneal space extending to the symphysis pubis. The drainage tube was connected to negative pressure suction to facilitate drainage of serous exudate and prevent seroma formation. The peritoneum was closed continuously with absorbable sutures (Figure 3F).

Postoperative care consisted of intravenous cefazolin and crystalloid infusion. On postoperative day (POD) 1, the preperitoneal drain collected 70 mL of serosanguinous fluid, with daily output decreasing to <20 mL by POD 3. The patient initiated oral intake on POD 2 with successful return of bowel function (flatus passage). The preperitoneal drain was removed on POD 5 following confirmation of minimal output (<10 mL/24 h). Discharge occurred on POD 6 after meeting standardized criteria: afebrile status, independent ambulation, and pain control (VAS score ≤ 2). During 18-month follow-up assessments including physical examination and dynamic ultrasound, no evidence of recurrence was documented. The patient reported satisfaction scores of 9/10 on the Carolinas Comfort Scale for mesh-related quality of life.

## Discussion

De Garengeot hernia, defined by the incarceration of the appendix within a femoral canal hernia sac, is a rare clinical entity. Since its initial description in 1731, fewer than 500 cases have been documented globally (1). This case demonstrates the feasibility of a single-stage laparoscopic TAPP combined with appendectomy and biologic mesh reinforcement – an approach that integrates minimally invasive surgical principles with the regenerative advantages of biologic scaffolds. To our knowledge, this represents the first reported application of biologic mesh in laparoscopic TAPP for this condition, simultaneously addressing acute appendicitis and femoral hernia reconstruction through a unified minimally invasive strategy.

### Diagnostic challenges and preoperative imaging

De Garengeot hernia poses significant diagnostic challenges due to non-specific symptoms overlapping with inguinal hernia and soft tissue infections. In this case, the patient exhibited a tender, irreducible right inguinal mass. CT was pivotal in identifying appendiceal incarceration within the femoral canal, demonstrating characteristic findings of appendiceal wall thickening and periappendiceal fat stranding – features consistent with prior studies validating CT as the gold standard for diagnosis (9). While Gómez-Portilla et al. reported CT sensitivity of 70% versus 12.5% for ultrasonography in preoperative identification (3), both modalities in this case successfully localized the appendix as the herniated content. This discrepancy underscores two critical considerations: (1) the diagnostic value of multimodal imaging integration, and (2) the necessity for context-dependent interpretation of reported sensitivity rates across heterogeneous cohorts. Physical examination findings complemented imaging: erythema over the hernia [present in 33.3% of cases (1)] combined with irreducible mass and localized tenderness formed a diagnostic triad that may enhance preoperative recognition accuracy.

### Advantages of laparoscopic TAPP

Laparoscopic TAPP offers multidimensional advantages in managing De Garengeot hernia, particularly in high-risk surgical candidates. Its minimally invasive nature permits direct visualization of hernia contents, enabling precise assessment of appendiceal viability and identification of concurrent intra-abdominal pathologies. Notably, Ikram et al. (10) described a case where preoperative CT failed to detect appendiceal incarceration; however, intraoperative laparoscopy revealed entrapment within the femoral ring, thereby preventing diagnostic oversight. This approach also facilitates real-time evaluation of appendiceal inflammation severity, guiding evidence-based decisions on appendectomy necessity and mesh selection (3).

The integration of laparoscopic appendectomy with TAPP allows single-stage management, eliminating the need for hybrid open-laparoscopic techniques or delayed interventions (11). Even in patients with previous inguinal hernia repairs, laparoscopic TAPP effectively manages adhesions and provides comprehensive femoral defect coverage, thereby minimizing recurrence (3, 12). The minimally invasive approach minimizes femoral canal dissection, reducing risks of chronic postoperative inguinodynia and iatrogenic nerve injury.

Emerging evidence supports laparoscopic preservation of non-inflamed appendices in selected cases. Gómez-Portilla et al. (3) described laparoscopic TAPP repair with appendiceal preservation in a patient without signs of inflammation, maintaining normal appendiceal function. Imataki et al. (13) reported two cases with preserved appendix viability and no recurrence or subsequent appendicitis during 18-month follow-up. This conservative strategy requires strict adherence to intraoperative viability assessment protocols, including 20-min observation intervals for confirming appendiceal viability, to avoid unnecessary appendectomies.

In this case, the laparoscopic TAPP technique enabled complete reduction of the hernia sac and anatomically precise deployment of a biologic mesh over the myopectineal orifice. The keyhole technique – involving circumferential dissection around the round ligament to optimize mesh fixation – was adapted from standardized inguinal hernia repair protocols, demonstrating effective recurrence prevention. Intraoperative identification of appendiceal ischemia mandated appendectomy to eliminate risks of delayed perforation or septic complications.

### Rationale for biologic mesh selection

Long-term complications such as erosion, migration, infection, chronic pain, intestinal obstruction, and fistula formation are primarily linked to the utilization of permanent synthetic meshes, which are more prevalent in use compared to absorbable synthetic and biologic meshes (14). Biologic meshes exhibit superior anti-infective properties relative to non-absorbable synthetic counterparts (15), though their clinical adoption remains limited by cost considerations. In China’s healthcare system, the porcine-derived biologic mesh (Surgisis) utilized in this case costs 9,800 CNY (approximately 1,350 USD), with 50% reimbursement through national basic medical insurance, rendering it financially accessible for most patients. Importantly, biologic mesh application in contaminated surgical fields reduces hernia recurrence rates, decreases hospitalization costs and eliminates repeat surgery-related psychological burdens (16).

In the context of preventing recurrence of ventral hernia in high-risk patients, biologic meshes are often the preferred choice due to their lower risk of infection and fewer other mesh-related complications (17, 18). In the future, synthetic absorbable materials may offer even lower prices and excellent results. Notably, prior reports of biologic mesh utilization in De Garengeot hernia have been restricted to hybrid approaches combining laparoscopic appendectomy with open repair. Klipfel et al. (8) described post-appendectomy biologic mesh placement via open femoral repair, whereas Mushtaq et al. (7) applied similar techniques in contaminated femoral hernia cases. Our case innovatively integrates biologic mesh reinforcement within a laparoscopic TAPP and appendectomy, demonstrating its technical feasibility and expanding application boundaries in De Garengeot hernia management.

### Technical considerations

Systematic review evidence indicates that biologic mesh application limited to bridging techniques in ventral hernia repair is associated with significantly higher recurrence rates compared to reinforced closure methods (19). Based on these findings, after complete hernia sac reduction, we implemented a reinforced closure strategy involving femoral ring approximation with interrupted 3-0 polydioxanone sutures followed by biologic mesh overlay. The round ligament was preserved using the keyhole technique, with circumferential mesh fixation avoiding ligament compression. No recurrence was observed during 18-month follow-up, validating this approach’s efficacy.

The biologic mesh is desiccated in its packaged state. Hydration via saline immersion restores pliability, facilitating intraoperative manipulation. However, unlike synthetic meshes, it lacks inherent shape memory and demonstrates propensity for edge curling and migration without adequate fixation. Consequently, the mesh is fixated to the transversalis fascia with interrupted absorbable sutures to prevent edge displacement and curling. Sutures are tensioned to <1 N to minimize nerve compression risks, particularly in the lateral femoral cutaneous nerve territory, thereby preventing chronic neuropathic pain. This technique adheres to HerniaSurge Group guidelines recommending avoidance of permanent fixation in preperitoneal repairs for nerve entrapment prevention (20).

Biologic meshes may exacerbate inflammatory responses, and the massive release of inflammatory mediators lead to a significant increase in postoperative exudation, which is associated with the formation of clinically significant seromas (21). We placed a drainage tube between the mesh and the peritoneum, which, although it prolonged the hospital stay, prevented the formation of seromas caused by the rapid accumulation of exudate. The drainage tube may be removed when sustained drainage volume is <10 ml/24 h for two consecutive days, provided no signs of infection or fluid collection are present.

### Limitations and future directions

While this case demonstrates the feasibility of Laparoscopic TAPP with biologic mesh, larger cohorts are needed to validate recurrence rates and long-term mesh performance. Additionally, biologic meshes are more expensive than synthetic alternatives, necessitating cost-benefit analyses in resource-limited settings. The synergistic integration of novel material design and nanotechnology significantly enhances the anti-infective efficacy of mesh materials through pathogen-selective inhibitory mechanisms (22).

Future studies should compare outcomes of biologic versus synthetic meshes in laparoscopic De Garengeot hernia repairs and explore the role of enhanced recovery protocols to shorten hospitalization.

## Conclusion

This case report demonstrates that laparoscopic TAPP with biologic mesh reinforcement is a feasible and effective single-stage strategy for De Garengeot hernia repair, achieving simultaneous resolution of acute appendicitis and hernia defect closure. The biologic mesh conferred anti-infective advantages in a contaminated field, with no postoperative complications or recurrence observed over 18 months. These findings support its potential to reduce morbidity and optimize recovery. However, larger prospective studies are needed to confirm long-term efficacy and cost-effectiveness, particularly in resource-constrained settings.

## Data Availability

The original contributions presented in this study are included in this article/supplementary material, further inquiries can be directed to the corresponding author.
